# The role of Cathepsin S as a marker of prognosis and predictor of chemotherapy benefit in adjuvant CRC: a pilot study

**DOI:** 10.1038/bjc.2011.408

**Published:** 2011-10-11

**Authors:** J A Gormley, S M Hegarty, A O'Grady, M R Stevenson, R E Burden, H L Barrett, C J Scott, J A Johnston, R H Wilson, E W Kay, P G Johnston, S A Olwill

**Affiliations:** 1 Fusion Antibodies Ltd, Springbank Industrial Estate, Pembroke Loop Rd, Belfast BT17 0QL, Northern Ireland; 2 The Centre for Medical Education, School of Medicine, Dentistry and Biomedical Sciences, Queen's University Belfast, Belfast, Northern Ireland; 3 Department of Histopathology, Royal College of Surgeons in Ireland, Beaumont Hospital, Dublin, Ireland; 4 Centre for Public Health, School of Medicine, Dentistry and Biomedical Sciences, Queen's University Belfast, Belfast, Northern Ireland; 5 Molecular Therapeutics, School of Pharmacy, Queen's University Belfast, Belfast, Northern Ireland; 6 Centre for Infection and Immunity, Queen's University Belfast, Belfast, Northern Ireland; 7 Centre for Cancer Research & Cell Biology, School of Medicine, Dentistry and Biomedical Sciences, Queen's University Belfast, Belfast, Northern Ireland

**Keywords:** Cathepsin S, colorectal cancer, predictive, prognostic, recurrence-free survival

## Abstract

**Background:**

The aim of this pilot retrospective study was to investigate the immunohistochemical expression of Cathepsin S (CatS) in three cohorts of colorectal cancer (CRC) patients (*n*=560).

**Methods:**

Prevalence and association with histopathological variables were assessed across all cohorts. Association with clinical outcomes was investigated in the Northern Ireland Adjuvant Chemotherapy Trial cohort (*n*=211), where stage II/III CRC patients were randomised between surgery-alone or surgery with adjuvant fluorouracil/folinic acid (FU/FA) treatment.

**Results:**

Greater than 95% of tumours had detectable CatS expression with significantly increased staining in tumours compared with matched normal colon (*P*>0.001). Increasing CatS was associated with reduced recurrence-free survival (RFS; *P*=0.03) among patients treated with surgery alone. Adjuvant FU/FA significantly improved RFS (hazard ratio (HR), 0.33; 95% CI, 0.12–0.89) and overall survival (OS; HR, 0.25; 95% CI, 0.08–0.81) among 36 patients with high CatS. Treatment did not benefit the 66 patients with low CatS, with a RFS HR of 1.34 (95% CI, 0.60–3.19) and OS HR of 1.33 (95% CI, 0.56–3.15). Interaction between CatS and treatment status was significant for RFS (*P*=0.02) and OS (*P*=0.04) in a multivariate model adjusted for known prognostic markers.

**Conclusion:**

These results signify that CatS may be an important prognostic biomarker and predictive of response to adjuvant FU/FA in CRC.

Colorectal cancer (CRC) is one of the leading causes of death worldwide, with over one million new cases diagnosed every year in the developed world (Boyle and Levin, 2008). Improved therapeutic strategies are urgently required, in terms of novel targets, improved drug efficacy and more accurate clinical guidance for postoperative treatment (Walther *et al*, 2009).

The development of metastasis in the liver and lung are the primary causes of death in CRC. Proteases are thought to promote the invasive and metastatic potential of tumours through their ability to remodel the extracellular matrix (ECM) (Rao, 2003; Gocheva and Joyce, 2007). Several groups of proteases have been shown to participate in ECM remodelling, including the matrix metalloproteinases, serine proteases and cysteine cathepsin proteases (Egeblad and Werb, 2002; Laufs *et al*, 2006; Novinec *et al*, 2007; Obermajer *et al*, 2008).

The cysteine cathepsins have recently emerged as key players in several tumourigenic processes (Gocheva *et al*, 2006; Mohamed and Sloane, 2006). They are a family of lysosomal proteases with physiological functions in protein turnover and processing (Chapman *et al*, 1997). Increased cathepsin expression and activity has been linked to many malignancies including glioma (Flannery *et al*, 2003), breast (Vasiljeva *et al*, 2006, 2008; Sevenich *et al*, 2010) prostate (Fernandez *et al*, 2001) and pancreatic cancer (Gocheva *et al*, 2006). In addition, despite their usual lysosomal localisation, they have been shown to be secreted and associated with the cell surface of tumour cells implying an extracellular role in cancer (Koblinski *et al*, 2002; Cavallo-Medved *et al*, 2003; Roshy *et al*, 2003).

Cathepsin S (CatS) distinguishes itself from many other family members with a restricted normal tissue expression, found mainly in lymphatic tissue and cells of monocyte/macrophage lineage, where it plays a key role in MHC class II presentation through invariant chain degradation (Kirchke *et al*, 1989; Bania *et al*, 2003). CatS has been found to be upregulated and linked with disease aggressiveness in several tumour types (Fernandez *et al*, 2001; Lindahl *et al*, 2009; Paraoan *et al*, 2009; Xu *et al*, 2009) and is of independent prognostic value in glioblastoma (Flannery *et al*, 2006). A number of previous studies have suggested that CatS promotes invasion and neoangiogenesis through ECM degradation and release of matrix-derived growth factors that drive the angiogenic switch (Shi *et al*, 2003; Joyce *et al*, 2004; Wang *et al*, 2006). In agreement with these mechanistic findings, we have recently demonstrated that specific inhibition of CatS by an antibody, Fsn0503, could attenuate CRC cell invasion *in vitro* and significantly reduce colorectal xenograft tumour growth (Burden *et al*, 2009) Although these recent *in vitro* and *in vivo* findings suggest that CatS has an important role in CRC pathogenesis, the evaluation of its clinical significance in CRC patient samples has not been performed to date.

In this study we aimed to evaluate the prevalence of CatS expression in primary and metastatic CRC tissue and to investigate its potential association with histopathological features and clinical outcomes. In order to achieve this we performed a pilot retrospective analysis of three cohorts of CRC patient samples (*n*=560); those from the Northern Ireland (NI) CRC Adjuvant Chemotherapy Trial, the Beaumont Hospital Dublin cohort and the US Biomax CO6161 cohort.

Due to local and ethical constraints, different levels of patient data were available for each cohort and associations were investigated accordingly. The NI Adjuvant trial cohort contained matched normal tissue and survival data, both for a group of patients treated with surgery alone and a group of patients treated with adjuvant fluorouracil/folinic acid (FU/FA) and therefore was the primary data set in the study. The Beaumont Hospital cohort contained lymph node metastatic tissue for comparison with matched primary tumour tissue. The US Biomax cohort was selected to supplement the NI adjuvant trial and Beaumont Hospital cohorts for prevalence information and for investigating associations with disease stage and grade.

## Patients and methods

### Patient samples

Three cohorts of patient samples were analysed for expression of CatS using CRC tissue microarrays (TMA). The NI adjuvant trial cohort was the primary data set; it consisted of 211 cases of matched CRC and adjacent normal tissue (four replicate cores/case) taken from the same patient, with clinical outcome information available. The Beaumont Hospital cohort consisted of 70 cases of Dukes C colorectal adenocarcinomas (12 replicate cores/case with 4 each from superficial, mid and deep areas of the tumour) and matched lymph node metastatic tissue (four replicate cores/case) taken from the same patient, which were retrieved from the pathology files at Beaumont Hospital, Dublin, from 2004 to 2009. The CO6161 TMA, obtained from US Biomax (Rockville, MD, USA), which consisted of 296 cases of CRC (two replicate cores/case), was used to assess prevalence and associations with disease stage and grade. All samples were taken under the appropriate local ethical and regulatory guidance with full consent from all patients. Cohort information is summarised in Table 1.

### NI CRC adjuvant chemotherapy trial

The NI CRC adjuvant chemotherapy trial was designed as a randomised controlled phase III study to compare 16 weeks of De Gramont schedule FU/FA adjuvant therapy to observation alone, following potentially curative surgery (McDermott *et al*, 2003; McLornan *et al*, 2010). A total of 254 patients with stages II and III CRC were recruited in 1994–1997 from hospitals throughout NI. Tissues were obtained from the initial resection specimen. There was full approval from the local research ethics committee and all involved hospitals, and all patients gave consent for the use of their specimens in research, according to the Declaration of Helsinki. In arm 1, protocol-defined follow-up alone occurred. In arm 2, 8 cycles of intravenous FA 200 mg m^−2^ as a 2-h infusion followed by bolus FU 400 mg m^−2^ and 22-h infusion FU 400 mg m^−2^ for 2 consecutive days every 2 weeks were used. Rectal cancer patients received postoperative adjuvant radiotherapy as clinically indicated. Patient age, sex, tumour stage and site were well balanced between arms. Median follow-up was 6.8 years. Of the 254 patients enrolled in study, only 211 were included in final IHC analysis; 42 cases could not be scored due to lack of availability of tissue or insufficient tissue.

### Immunohistochemistry

All TMAs were stained at the same time under identical conditions. Full experimental details for all immunohistochemical staining are provided in the Supplementary data.

### Scoring

All cases were independently scored by two investigators (SMH, JAG) who were blinded to clinical data. Tumour and normal colonic mucosa samples were scored as 0, 1+, 2+ or 3+ for intensity of staining. In order to increase reliability and repeatability, this scoring regime was agreed by both investigators, based on observation of the staining, before independent scoring. In any cases of discordance (4%), cores were reviewed until a consensus was reached and scoring was further spot-checked by a third investigator (EWK). Polarisation to the apical or basal membrane was noted. Modal scores were determined for replicates of each case. Scores were reclassified as low (0 and 1+), moderate (2+) and high (3+) expression for statistical analysis. In cases where a biphasic or multiphasic distribution of staining occurred within the tumours or normal colonic mucosa, the intensity that covered the higher extent of the core was chosen. Criteria were set to determine if sufficient tissue was available for reliable determination of CatS score and cases were excluded if insufficient tissue was available

### Statistics

Ordinal regression was used to evaluate the significance of difference in CatS expression level between matched tumour and normal tissue and matched metastatic and primary tumour tissue taken from the same specimen. Ordinal regression was also used to test for association between CatS expression and disease stage or grade. Stage and grade were considered as linear variables in these analyses. Association between CatS level and lymphovascular invasion (LVI) status or tumour site was analysed using the Pearson's *χ*^2^-test. In survival analyses, the primary clinical outcome variables were recurrence-free survival (RFS), defined as the time from randomisation to radiologically or histologically proven recurrence of CRC; and overall survival (OS), defined as the time from randomisation to CRC-related death. The aim of this retrospective study was to specifically investigate any potential links between CatS expression and cancer progression to death and therefore non-CRC-related death resulted in censoring of data at that time point. The follow-up time was censored at death from any cause, loss to follow-up or at 100 months. Survival times according to different variables were compared by the Kaplan–Meier method and log-rank test. Univariate and multivariate hazard ratios (HR) were calculated using Cox proportional hazards modelling. CatS expression was treated as a continuous linear variable in the multivariate analysis. Tests of proportionality, based on plotting of partial residual values for each covariate, were run to verify the Cox Proportional Hazards assumption. All reported *P*-values were two-sided and *P*-values of less that 0.05 were considered to be statistically significant. Statistics were performed using SPSS 17.0 (SPSS Inc, Chicago, IL, USA).

## Results

### Specific immunohistochemical detection of CatS

**Figure 1:**
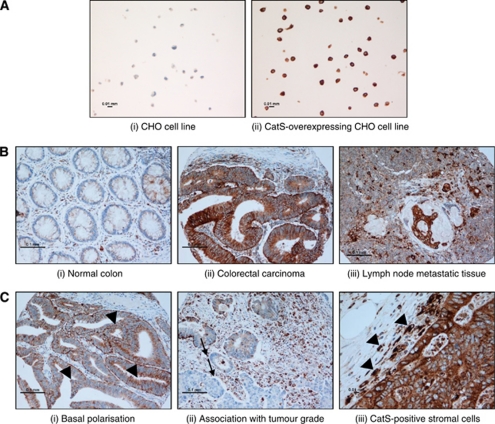
Representative images of CatS expression patterns in cell lines and patient samples. CatS-specific staining is brown and nuclear counterstaining is blue. (**A**) Parental CHO- and CatS-CHO-overexpressing cell lines where CatS-specific staining is evident in the overexpressing line only. (**B**) Normal colonic mucosa, where a finely granular pattern is observed; and in colorectal carcinoma and lymph node metastatic tissue, where an increase in expression is evident. (**C**) Distinct patterns of expression were observed in tumours such as basal epithelial polarisation, indicated with arrow heads; loss of expression concomitant with loss of differentiation, indicated with arrows; and intense CatS expression in tumour-associated cells, indicated with arrow heads. The scale can be found on images.

A monoclonal antibody to human CatS, Fsn0503, was used for immunohistochemical staining of all samples herein. The specificity of Fsn0503 over other cathepsin family members has previously been demonstrated (Burden *et al*, 2009). Furthermore, IHC staining of wild-type Chinese hamster ovary (CHO) cells and CatS-overexpressing CHO cells for CatS expression showed staining in the overexpressing CHO cell line only, demonstrating specificity of the antibody by IHC (Figure 1A). Fsn0503 recognises both the zymogen and active forms of the protease by the western blot (data not shown).

### Prevalence of CatS in colorectal carcinoma and metastatic tissue

CatS expression in tumour tissue was assessed across all cohorts (*n*=560). Low expression was found in 31% of cases, moderate expression in 52% and high expression in 17% of cases. A low to negligible level of finely granular cytoplasmic staining was displayed in normal colonic mucosa, whereas an intense, coarsely granular cytoplasmic staining pattern was observed in tumour samples and matched metastatic tissue (Figure 1B). Epithelial cells in 60% of the tumours contained CatS diffusely expressed throughout the cytoplasm but in the remainder of tumours, the expression was alternatively polarised to either the apical or basal pole of the epithelium (Figure 1C). A decrease in CatS staining with transition from moderately to poorly differentiated tumour was observed (Figure 1C). Additionally it is of note that subpopulations of stromal cells, possibly tumour-associated macrophages, stained positive for CatS (Figure 1C).

CatS expression in matched pairs of tumour and adjacent normal tissue could be compared for 175 out of 211 cases in the NI CRC cohort, with remaining cases excluded due to insufficient tissue. A 1.3-fold increase in CatS expression was found in tumours compared with normal tissues (*P*<0.001; Supplementary Table 5). Matched lymph node metastatic tissue was available for 67 samples of CRC in the Beaumont Hospital cohort. CatS was found to be expressed in >95% cases of metastatic tumour tissue found in lymph nodes, with a significantly higher (1.2-fold) expression in the primary tumour tissue compared with involved nodal tissue (*P*=0.03; Supplementary Table 5).

### Association of CatS expression with clinicopathological traits

We investigated the potential association of CatS expression with well-known clinicopathological features of disease stage, grade, tumour site and LVI status. A significant association of increasing CatS expression with decreasing tumour grade was noted (*P*=0.005; Table 2); however, no correlation between CatS expression and other pathological features were found.

### Association of CatS expression with survival

Of the 254 patients enrolled in the NI CRC trial, 211 were included in the survival analyses, 106 in the surgery alone (‘untreated’) group and 105 in the adjuvant FU/FA-treated (‘treated’) group. RFS and OS were monitored with a median follow-up time of 6.8 years. In all, 43 of the 57 patients (75%) with rectal cancer received adjuvant postoperative radiation therapy as per clinical guidelines extant at the time of the trial. Seven cases (16%) of rectal cancer recurred primarily locally, which is comparable to standard post-surgical incidence rates for this time period (Kapiteijn *et al*, 2001). As the relative levels of CatS (from low to moderate to high) were found to be similarly distributed among rectal patients compared with the entire patient cohort, rectal cases were included in the analysis.

**Figure 2:**
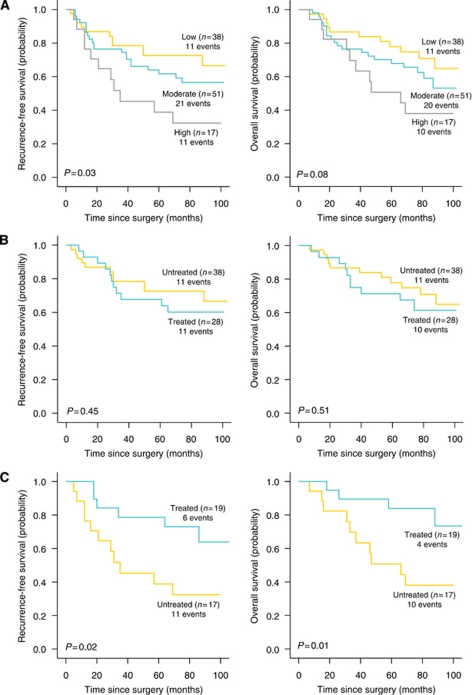
Kaplan–Meier analysis of 8-year RFS and 8-year OS in the NI Cancer Centre cohort according to (**A**) CatS expression level in the untreated group of patients; (**B**) treatment status in patients with low CatS expression and; (**C**) treatment status in patients with high CatS expression. Log-rank *P*-values are shown. Abbreviations: events, incidents of disease recurrence or death due to CRC; n, sample size.

In a pooled analysis for all patients, by the end of the follow-up period 43% of patients had died, 34% from CRC, and disease had recurred in 37% of cases. There was no evidence of an association between CatS expression and RFS or OS in this pooled group. Among untreated patients CatS expression was associated with poor 8-year RFS (*P*=0.03; Figure 2A), with an estimated HR of 1.72 (95% CI, 1.13–2.66; *P*=0.01, Table 3). This trend, although apparent, was not found to be significant for 8-year OS (*P*=0.08; Figure 2A), with an estimated HR of 1.62 (95% CI, 1.05–2.51; *P*=0.03; Table 3). Among treated patients, there was no association between CatS and RFS or OS.

### Association of CatS expression with benefit of adjuvant FU/FA

There was a nonsignificant trend towards improved RFS and OS in the 105 treated patients compared with the 106 untreated patients. A significant interaction was found between CatS expression and the RFS benefit from adjuvant FU/FA (*P*=0.03) and this trend remained upon stratification for disease stage (*P*=0.01). Similarly, there was a trend for OS (*P*=0.02), which remained when stratified by stage (*P*=0.01).

Among the 36 patients (17%) with high CatS expression, there was a significant benefit from treatment (*P*=0.02 for RFS and *P*=0.01 for OS; Figure 2C). The 8-year RFS HR was 0.33 (95% CI, 0.12–0.89; *P*=0.03) and the 8-year OS HR was 0.25 (95% CI, 0.08–0.81; *P*=0.02; Table 3). There was no evidence of treatment benefit in the 52% of patients with moderate CatS expression; the 8-year RFS HR was 0.69 (95% CI, 0.37–1.30; *P*=0.25) and the 8-year OS HR was 0.70 (95% CI, 0.37–1.34; *P*=0.28; Table 3). In the 31% of patients with low CatS expression, again there was no evidence of treatment benefit. The 8-year RFS HR was 1.34 (95% CI, 0.60–3.19; *P*=0.45) and the 8-year OS HR was 1.33 (95% CI, 0.56–3.15; *P*=0.52; Table 3).

In a multivariate model, adjusted for disease stage, LVI status and tumour site, the interaction between CatS expression and treatment status was significant for both RFS (HR 0.46; 95% CI, 0.24–0.90; *P*=0.02) and OS (HR 0.49; 95% CI, 0.50–0.96; *P*=0.04; Table 4). The trend held when stratified for stage; RFS HR was 0.45 (95% CI, 0.23–0.88; *P*=0.02) and OS HR was 0.46 (95% CI, 0.23–0.91; *P*=0.03; Supplementary Table 6). When dichotomised stage subgroups were interrogated independently, the trend only reached significance in the stage III subgroup. The low patient numbers in stage subgroups limited the power of these analyses. The estimated HR for the interactive term was 0.57 (95% CI, 0.20–1.61; *P*=0.29) for RFS and 0.45 (95% CI, 0.15–1.36; *P*=0.16; Supplementary Table 7) for OS for stage II and 0.38 (95% CI, 0.15–0.93; *P*=0.03) for RFS and 0.45 (95% CI, 0.18–1.12; *P*=0.09; Supplementary Table 7) for OS for stage III.

## Discussion

We have shown that CatS is expressed in 95% of cases of primary colorectal tumours and their related metastatic tissue, with significantly higher expression in tumours compared with matched normal colonic mucosa. We have also demonstrated that CatS is an independent prognostic marker of poor outcome and is predictive of improved response to adjuvant FU/FA treatment in this disease.

The trends observed are consistent with the reported pro-tumourigenic role of cathepsins in cancer (Gocheva *et al*, 2006). Cathepsins are potent degradative enzymes whose normal restricted proteolytic activity is altered by neoplastic cells resulting in secretion into the tumour microenvironment and cleavage of ECM component proteins (Obermajer *et al*, 2008). This ECM remodelling in turn facilitates tumour growth, angiogenesis, invasion and metastasis (Joyce *et al*, 2004; Gocheva *et al*, 2006; Bell-McGuinn *et al*, 2007). Cathepsins have been shown to be upregulated and aberrantly expressed and linked with prognosis in several cancers (Foekens *et al*, 1998; Mohamed and Sloane, 2006). Most notably, cathepsins B and L have both been linked with unfavourable outcome in CRC (Campo *et al*, 1994; Troy *et al*, 2004).

CatS has recently emerged as a relevant biomarker in cancer with reports of association with poor prognosis in glioblastoma (Flannery *et al*, 2006) and with disease aggression in uveal melanoma (Paraoan *et al*, 2009), hepatocellular carcinoma (Xu *et al*, 2009) and prostate carcinoma (Fernandez *et al*, 2001; Lindahl *et al*, 2009). However, this is the first study to investigate the clinical significance of CatS in CRC. Here we have demonstrated that CatS is upregulated in both primary CRC and related metastatic nodal tissue and displays a polarised pattern of expression that could be suggestive of secretion and action in the tumour microenvironment. Interestingly we find a significantly higher expression level of CatS in primary tumour compared with lymph node metastatic tissue, which may imply a more important role for the enzyme in promoting tumour cell invasion and dissemination from the primary site. We also observe an association between increasing CatS expression and decreasing tumour grade, perhaps also reflecting the importance of CatS in early tumour development.

We also show that CatS is independently prognostic for reduced 8-year RFS and demonstrates a similar prognostic trend toward reduced 8-year OS in a patient group receiving no post-surgical chemotherapy. These trends were independent of disease stage and suggest that stratification based on CatS expression may aid in identification of low- and high-risk patient groups still in a potentially curative treatment setting. Furthermore, given the observed negative correlation between CatS expression and tumour grade, stratification based on CatS may help to identify high-risk patient subgroups that may otherwise be considered as low risk due to a well-differentiated histopathology (Compton, 2007). Taken together with data from murine studies, our data support a role for CatS in tumourigenesis and support its potential both as a biomarker for disease prognosis and as a therapeutic target in CRC (Small *et al*, 2011).

In an adjuvant FU/FA-treated group of patients, CatS was found to correlate with increasing response to treatment. This interaction between CatS and treatment benefit was significant in a multivariate model adjusted for other known prognostic markers and although not reaching significance, the trend was observed when stage subgroups (II and III) were interrogated independently. The data suggest that although patients with high CatS are at higher risk of recurrence, they can benefit most from adjuvant FU/FA therapy. In contrast patients whose tumours express low CatS levels have a lower risk of recurrence and may be harmed by treatment. Our observations come from a clinical trial conducted in an era of fluoropyrimidine monotherapy use in adjuvant therapy of CRC. These results require further substantiation using prospective clinicopathological data sets from trials employing combination of fluoropyrimidine and oxaliplatin as adjuvant therapy for high-risk stage II and stage III CRC.

Given the continuing clinical quandary as to whether patients with stage II CRC should receive chemotherapy or not, these trends are intriguing (Graziano and Cascinu, 2003; Benson III *et al*, 2004). Chemotherapy is generally indicated for high-risk stage II patients, however, absolute OS benefits remain very low (<5%) (Benson III, 2006). Currently, there is much endeavour to find novel biomarkers and gene signatures that are predictive of response to chemotherapy in order to avoid the unnecessary debilitating side-effects of ineffective treatment (Graziano and Cascinu, 2003; Braun *et al*, 2008; Zlobec and Lugli, 2008; Koopman *et al*, 2009; Walther *et al*, 2009). Our data suggest that stratification, which uses CatS as a biomarker may potentially facilitate this. To date, many of the potential predictive markers of FU response that have been investigated have proven insufficient for inclusion in clinical practice and new markers are urgently needed (Ribic *et al*, 2003; Kim *et al*, 2007; Bertagnolli *et al*, 2009). This underlines the importance of further investigation into CatS as a predictive marker, with respect to adjuvant treatment with fluoropyrimidines alone or with oxaliplatin.

In conclusion, our data suggests the potential utility of CatS as a prognostic indicator in an untreated group of patients and as a novel predictive biomarker of response to fluoropyrimidines. It also suggests a therapeutic rationale for targeting this enzyme in CRC. It is important to underline that this is a pilot retrospective study and where survival trends were consistent between OS and RFS for stage stratifications, significance was not always attained when stage subgroups were analysed independently, possibly due to low patient numbers. In addition, it is important to emphasise that the mechanistic relevance of expression patterns, such as polarisation and association with tumour grade, need further investigation. The observations regarding CatS will require robust confirmation in a larger cohort of patients, as part of prospective studies using fluoropyrimidines both with and without oxaliplatin, before consideration for inclusion in clinical evaluation.

## Figures and Tables

**Table 1:** Clinicopathological information for NI CRC adjuvant chemotherapy trial, Beaumont Hospital and US Biomax cohorts

	***n* (%)**	**Beaumont hospital**	**NI CRC adjuvant chemotherapy trial**	**US Biomax CO6161**
Patients	560	70	211	279
Matched normal samples	175	0	175	0
Matched lymph node samples	67	67	0	0
Median (range) age (years)		—	65 (35–80)	57 (26–86)
				
*Gender*				
Male	353 (63)	50	126	177
Female	207 (37)	20	85	102
				
*Tumour site* a				
Proximal	114 (41)	33	81	
Distal	91 (32)	21	70	—
Rectum	73 (26)	16	57	
Synchronous	3 (1)	—	3	
				
*Stage (TNM)*				
I	22 (7)	0	0	22
II	318 (53)	0	136	182
III	206 (37)	64	75	67
IV	14 (3)	6	—	8
				
*Grade*				
I	46 (8)	0	19	27
II	403 (72)	60	160	183
III	90 (16)	10	24	56
Unknown	21 (4)	—	8	13
				
*LVI status*				
Y	79 (28)	34	45	
N	127 (45)	16	111	—
Unknown	75 (27)	20	55	

Abbreviations: CRC=colorectal cancer; LVI=lymphovascular invasion; NI=Northern Ireland; (—)=Information was not available where indicated.

a Proximal: caecum, ascending colon, hepatic flexure, transverse colon; distal: descending colon, sigmoid colon; rectal: rectosigmoid colon, rectum.

**Table 2:** Association of CatS expression with clinicopathological features

		**Antigen expression level**			
	***n* (%)**	**Low (%)**	**Moderate (%)**	**High (%)**	***P***
*Grade*	539				
Well differentiated (I)	46 (8)	16 (35)	17 (37)	13 (28)	
Moderately differentiated (II)	403 (75)	142 (35)	180 (45)	81(20)	0.005^a^
Poorly differentiated (III)	90 (17)	46 (51)	32 (36)	12 (13)	
					
*Stage (TNM)*	558				
I	22 (4)	13 (59)	7 (32)	2 (9)	
II	318 (57)	120 (38)	141 (44)	57 (18)	0.15^a^
III	206 (37)	72 (35)	87 (42)	47 (23)	
IV	14 (3)	7 (50)	5 (36)	2 (14)	
					
*Tumour site* c	278				
Proximal	114 (41)	34 (30)	59 (52)	21 (18)	
Distal	91 (33)	26 (29)	47 (51)	18 (20)	0.98^b^
Rectum	73 (26)	23 (32)	35 (48)	15 (20)	
					
*LVI status*	206				
Yes	78 (38)	23 (30)	37 (47)	18 (23)	0.53^b^
No	128 (62)	37 (29)	69 (54)	22 (17)	

Abbreviations: CatS=Cathepsin S; LVI=lymphovascular invasion.

Association of CatS with disease stage and tumour grade was assessed in all cohorts. In all, 20 cases of unknown grade were removed from the grade analysis. Association of CatS with tumour site and LVI status was assessed in the Beaumont Hospital and NI Cancer Centre cohorts only. Three cases of synchronous location were removed in the tumour site analysis and 55 cases of unknown LVI status were removed from the LVI analysis.

a Calculated *P*-values are from ordinal regression.

b Calculated *P*-values are from Pearson's *χ*^2^-tests.

c Proximal: caecum, ascending colon, hepatic flexure, transverse colon; distal: descending colon, sigmoid colon; rectal: rectosigmoid colon, rectum.

**Table 3:** Univariate analysis for 8-year RFS and OS in (**A**) all patients according to CatS or treatment status, (**B**) treatment subgroups according to CatS and (**C**) CatS-stratified subgroups according to treatment

	**8-year RFS**				**8-year OS**			
	**%**	**HR**	**95% CI**	***P***	**%**	**HR**	**95%CI**	***P***
**(A)**								
CatS (*n*=211)								
Low	63				63			
Moderate	63	1.22	0.88–1.69	0.23	62	1.11	0.79–1.55	0.54
High	54				62			
Treatment (*n*=211)								
Untreated	56	0.75	0.48–1.17	0.20	55	0.71	0.45–1.13	0.15
Treated	64				68			
								
**(B)**								
Untreated (*n*=106)								
Low	67				65			
Moderate	57	1.72	1.13–2.66	0.01	53	1.62	1.05–2.51	0.03
High	32				38			
Treated (*n*=105)								
Low	60				61			
Moderate	68	0.84	0.51–1.39	0.50	70	0.74	0.44–1.26	0.27
High	64				73			
								
**(C)**								
Low (*n*=66)								
Untreated	67	1.34	0.60–3.19	0.45	65			
Treated	60				61	1.33	0.56–3.15	0.52
Moderate (*n*=109)								
Untreated	57	0.69	0.37–1.30	0.25	53	0.70	0.37–1.34	0.28
Treated	68				70			
High (*n*=36)								
Untreated	32	0.33	0.12–0.89	0.03	38	0.25	0.08–0.81	0.02
Treated	64				73			

Abbreviations: CatS=Cathepsin S; CI=confidence interval; OS=overall survival; treated=patients treated with adjuvant fluorouracil/folinic acid; RFS**=**recurrence-free survival; untreated=patients receiving no adjuvant chemotherapy.

**Table 4:** Multivariate Cox regression analysis of 8-year RFS and OS

	**8-year RFS**			**8-year OS**		
	**HR**	**95% CI**	***P***	**HR**	**95% CI**	***P***
CatS expression	1.81	1.17–2.82	0.08	1.47	0.96–2.26	0.08
Treatment status	1.25	0.59–2.66	0.56	1.22	0.57–2.61	0.60
Treatment status ∝ CatS expression^a^	0.46	0.24–0.90	0.02	0.49	0.50–0.96	0.04
Stage	3.12	1.96–4.95	<0.001	2.79	1.72–4.53	<0.001
LVI (yes *vs* no/NOS)	1.90	1.19–3.03	0.007	2.04	1.24–3.35	0.005
Tumour site (proximal *vs* distal)^b^	2.00	1.11–3.61	0.02	2.00	1.08–3.71	0.03
Tumour site (rectal *vs* distal)^b^	2.36	1.28–4.36	0.006	2.10	1.11–3.96	0.03

Abbreviations: CatS=Cathepsin S; CI=confidence interval; HR=hazard ratio; LVI=lymphovascular invasion; NOS=not otherwise specified; OS=overall survival; RFS=recurrence-free survival.

The tumour site was considered as a categorical variable comparing proximal and rectal locations to distal. Three cases of synchronous location were excluded from the model.

a Interactive term for CatS expression and treatment status.

b Proximal: caecum, ascending colon, hepatic flexure, transverse colon; distal: descending colon, sigmoid colon; rectal: rectosigmoid, rectum.
