# Supplementary material for: The role of Cathepsin S as a marker of prognosis and predictor of chemotherapy benefit in adjuvant CRC: a pilot study
Source: Br J Cancer. 2011 Oct 11;105(10):1487–94. doi: 10.1038/bjc.2011.408 (PMC3242524; doi:10.1038/bjc.2011.408)
Supplement: Supplementary Information [file bjc2011408x1.doc]

**Supplementary Data**

**Methods**

**Immunohistochemistry**

Deparaffinization, antigen retrieval and immunohistochemistry (IHC) were performed on paraffin-embedded 4 µm tissue microarray sections and on whole sections using an automated IHC platform (Bond Max^TM^, Leica Microsystems, Newcastle, U.K.). Anti-CatS (monoclonal antibody (clone 1E11, Fusion Antibodies) was diluted to 1/1000 in antibody diluent (Refine cat#AR9352). A polymer-based detection system (Refine cat#DS9800) was used with 3’,3-Diaminobenzidine (DAB) as the chromogen. The positive controls included a sample of colonic adenocarcinoma and inflamed tonsil and the negative control was performed on all cases by excluding the primary antibody.

**Tissue microarray construction**

For NI Cancer Centre cohort, arrays were constructed as described (McLornan *et al*, 2010). For Beaumont Hospital cohort, using a Beechers manual tissue arrayer, a tissue microarray (TMA) was constructed using a 0.6mm punch.

**Statistics**

A Goodness of fit test was carried out to check the assumptions of the Ordinal Regression model (McCullough, 1980).

**Results**

|  | **Table 5.** Ordinal Regression analysis to compare CatS expression levels in normal colon, primary tumour tissue and lymph node metastatic tissue | | | | |  |
| --- | --- | --- | --- | --- | --- | --- |
|  |  | **N** | ***P*** | **Estimate (95% CI)** | **Wald** |  |
|  | Primary tumour vs normal colon | 176 | <0.001 | 2.054 (1.444 – 2.664) | 43.533 |  |
|  | Lymph node metastases vs. primary tumour | 70 | 0.030 | -1.333 (- 2.54 - -0.126) | 4.682 |  |
|  |  | | | | |  |

|  | **Table 6**. Multivariate Cox Regression analysis of 8-year RFS and OS after Stratification for Disease Stage | | | | | | | | |  |
| --- | --- | --- | --- | --- | --- | --- | --- | --- | --- | --- |
|  |  |  | **8-year RFS** | | |  | **8-year OS** | | |  |
|  |  |  | **HR** | **95% CI** | ***P*** |  | **HR** | **95% CI** | ***P*** |  |
|  | CatS expression |  | 1.83 | 1.18 to 2.85 | 0.008 |  | 1.52 | 0.98 to 2.34 | 0.06 |  |
|  | Treatment status |  | 1.29 | 0.61 to 2.76 | 0.51 |  | 1.29 | 0.60 to 2.77 | 0.51 |  |
|  | *Treatment status µ CatS expression |  | 0.45 | 0.23 to 0.88 | 0.02 |  | 0.46 | 0.23 to 0.91 | 0.03 |  |
|  | LVI- (Yes vs No/ NOS) |  | 1.88 | 1.17 to 3.00 | 0.009 |  | 2.06 | 1.25 to 3.39 | 0.04 |  |
|  | †Tumour site (Proximal vs Distal) |  | 1.99 | 1.10 to 3.59 | 0.02 |  | 2.05 | 1.10 to 3.82 | 0.02 |  |
|  | †Tumour site (Rectal vs Distal) |  | 2.38 | 1.29 to 4.42 | 0.006 |  | 2.16 | 1.14 to 4.09 | 0.02 |  |
|  | Tumour site was considered as a categorical variable comparing Proximal and Rectal locations to Distal. Three cases of synchronous location were excluded from the model.  **,* interactive term for CatS expression and treatment status.  †Proximal (caecum, ascending colon, hepatic flexure, transverse colon), Distal (descending colon, sigmoid colon), Rectal (rectosigmoid, rectum).  Abbreviations: CI, confidence interval; NOS, not otherwise specified. | | | | | | | | |  |

|  | **Table 7.** Multivariate Cox Regression analysis of 8-year RFS and OS for stage subgroups | | | | | | | | |  |
| --- | --- | --- | --- | --- | --- | --- | --- | --- | --- | --- |
|  |  |  | **8-year RFS** | | |  | **8-year OS** | | |  |
|  | **Stage II** |  | **HR** | **95% CI** | ***P*** |  | **HR** | **95% CI** | ***P*** |  |
|  | CatS expression |  | 2.18 | 1.07 to 4.47 | 0.03 |  | 2.25 | 1.09 to 4.68 | 0.03 |  |
|  | Treatment status |  | 1.47 | 0.40 to 5.38 | 0.56 |  | 1.43 | 0.38 to 5.41 | 0.60 |  |
|  | *Treatment status µ CatS expression |  | 0.57 | 0.20 to 1.61 | 0.29 |  | 0.45 | 0.15 to 1.36 | 0.16 |  |
|  | LVI- (Yes versus No/ NOS) |  | 1.57 | 0.78 to 3.15 | 0.20 |  | 2.11 | 1.01 to 4.38 | 0.05 |  |
|  | †Tumour site (Proximal versus Distal) |  | 1.20 | 0.51 to 2.81 | 0.67 |  | 1.18 | 0.48 to 2.87 | 0.72 |  |
|  | †Tumour site (Rectal versus Distal) |  | 1.95 | 0.82 to 4.63 | 0.13 |  | 1.86 | 0.75 to 4.63 | 0.18 |  |
|  |  |  |  |  |  |  |  |  |  |  |
|  | **Stage III** |  | **HR** | **95% CI** | ***P*** |  | **HR** | **95% CI** | ***P*** |  |
|  | CatS expression |  | 1.59 | 0.90 to 2.83 | 0.11 |  | 1.16 | 0.66 to 2.02 | 0.61 |  |
|  | Treatment status |  | 1.25 | 0.49 to 3.20 | 0.65 |  | 1.28 | 0.50 to 3.30 | 0.61 |  |
|  | *Treatment status µ CatS expression |  | 0.38 | 0.15 to 0.93 | 0.03 |  | 0.45 | 0.18 to 1.12 | 0.09 |  |
|  | LVI- (Yes versus No/ NOS) |  | 2.20 | 1.12 to 4.32 | 0.02 |  | 2.29 | 1.12 to 4.68 | 0.02 |  |
|  | †Tumour site (Proximal versus Distal) |  | 3.30 | 1.37 to 7.95 | 0.008 |  | 3.70 | 1.49 to 9.19 | 0.005 |  |
|  | †Tumour site (Rectal versus Distal) |  | 3.29 | 1.31 to 8.23 | 0.01 |  | 2.93 | 1.15 to 7.43 | 0.02 |  |
|  | Tumour site was considered as a categorical variable comparing Proximal and Rectal locations to Distal. Three cases of synchronous location were excluded from the model.  **,* interactive term for CatS expression and treatment status.  †Proximal (ascending colon, caecum, transverse colon), Distal (descending colon, sigmoid colon), Rectal (rectosigmoid, rectum).  Abbreviations: CI, confidence interval; NOS, not otherwise specified. | | | | | | | | |  |
